# Hypoxia promotes chemoresistance in acute lymphoblastic leukemia cell lines by modulating death signaling pathways

**DOI:** 10.1186/s12885-016-2776-1

**Published:** 2016-09-22

**Authors:** C. Petit, F. Gouel, I. Dubus, C. Heuclin, K. Roget, J. P. Vannier

**Affiliations:** 1 MERCI EA3829, Université de Rouen, Faculté de Médecine-Pharmacie, 22 Boulevard Gambetta, 76183 Rouen, France; 2 BioSIMS Technologies, 75 Route de Lyons la forêt, Seine BioPolis, 76000 Rouen, France; 3 Enterome, 94/96 avenue Ledru-Rollin, 75011 Paris, France

**Keywords:** ALL, Chemoresistance, Hypoxia, Methotrexate, Prednisolone, RPPA

## Abstract

**Background:**

Several studies show that bone marrow (BM) microenvironment and hypoxia condition can promote the survival of leukemic cells and induce resistance to anti-leukemic drugs. However, the molecular mechanism for chemoresistance by hypoxia is not fully understood.

**Methods:**

In the present study, we investigated the effect of hypoxia on resistance to two therapies, methotrexate (MTX) and prednisolone (PRD), in two cell models for acute lymphoblastic leukemia (ALL). To look for an implication of hypoxia in chemoresistance, cell viability, total cell density and cell proliferation were analyzed. Survival and death signaling pathways were also screened by “reverse phase protein array” (RPPA) and western blotting experiments conducted on selected proteins to confirm the results.

**Results:**

We found that hypoxia promotes chemoresistance in both ALL cell lines. The induction of drug-resistance by hypoxia was not associated with an increase in total cell density nor an increase in cell proliferation. Using RPPA, we show that chemoresistance induced by hypoxia was mediated through an alteration of cell death signaling pathways. This protective effect of hypoxia seems to occur via a decrease in pro-apoptotic proteins and an increase in anti-apoptotic proteins. The results were confirmed by immunoblotting. Indeed, hypoxia is able to modulate the expression of anti-apoptotic proteins independently of chemotherapy while a pro-apoptotic signal induced by a chemotherapy is not modulated by hypoxia.

**Conclusions:**

Hypoxia is a factor in leukemia cell resistance and for two conventional chemotherapies modulates cell death signaling pathways without affecting total cell density or cell proliferation.

**Electronic supplementary material:**

The online version of this article (doi:10.1186/s12885-016-2776-1) contains supplementary material, which is available to authorized users.

## Background

Oxygen levels in bone marrow are very heterogeneous with levels ranging from 1 to 7 % [1]. Several studies mention that microenvironment from BM and more specifically the hypoxia condition can promote the survival of leukemic cells. Hypoxia plays an important role in numerous physiological processes, including cell metabolism, cell survival, cell proliferation, angiogenesis and in pathological processes, like cancerogenesis, and metastasis [2–4]. Hypoxia is a negative prognostic and predictive factor due to its pathological features [5]. Indeed, recently it has been shown that hypoxia contributes to chemotherapy and radiotherapy resistance of leukemic cells and that hypoxic areas are associated with progression of leukemia [6, 7]. Leukemic cells from bone marrow of childhood acute lymphoblastic leukemia (ALL) have shown an overexpression of a key marker of hypoxia called hypoxia inducible factor (HIF-1α). In normoxic conditions, HIF-1α is degraded by 26S proteasome. Conversely, in hypoxic conditions, as often found in the microenvironment of bone marrow, HIF-1α is stabilized and dimerized with a HIF-1β subunit to activate the transcription of several genes involved in glucose metabolism, angiogenesis and cell survival [8]. Thus, HIF-1α activity is associated with increase of tumor progression and therapeutic resistance of leukemic cells and its stabilization correlates with poor prognosis of patients [9].

ALL is the most common form of pediatric cancer (approximately 35 % of childhood cancers). Due to progress in treatments, the survival rate has increased from 20 % in the 60’s to 70–90 % depending on the leukemia type. ALL is characterized by a deregulated proliferation process of a lymphoblastic population blocked at an immature stage in the BM. Several chemotherapies blocking the cell cycle and inducing cell death are currently available for ALL treatment such as vincristine and methotrexate [10]. Despite improved treatment protocols and a better management of leukemia patients, a significant number of patients relapse due to chemotherapy failure [11, 12]. Relapses of leukemia patients are due to the persistence of lymphoblastic cells that are resistant to treatment and already present at the diagnosis. The continued presence of these cells has already been linked to a disruption of the apoptotic pathways [13, 14], the development of malignant phenotypes and their chemoresistance [15], and a bad prognosis and increased risk of relapse [16]. Several explanation of relapse have been proposed. They include the persistence of blasts out of reach for chemotherapies, leukemic cells blocked in G0 phase, leukemic cells intrinsic drug resistance present at diagnosis and leukemic cells with acquired resistance [17]. Most of these relapses are due to deregulation of molecular mechanisms. Therefore, the study of intracellular signaling pathways is essential to understand chemoresistance in leukemia.

New techniques for proteomic studies such as reverse phase protein array (RPPA) have been developed to allow a straightforward screening of signaling pathways on several protein lysates. The RPPA method allows the measurement of protein expression levels and their post-translational states (cleavage, phosphorylation, ubiquitination) in a low volume of sample. RPPA has already been used to demonstrate the importance of several proteins and their post-translational modification in tumorogenesis [18, 19].

In our study, two different chemotherapies, methotrexate (MTX) and prednisolone (PRD), were used to evaluate the chemoresistance of leukemic cells. MTX is a folate analog used in the treatment of childhood B-ALL and auto-immune disease which is able to inhibit deoxyribonucleic acid (DNA) synthesis through thymidylate and purine nucleotides depletion [20]. The loss of DNA precursors by MTX induces DNA strand breakage and cell death [21, 22]. PRD is a glucocorticoid used to predict long term clinical outcome of ALL patients which diffuses passively into the cell and binds to glucocorticoid receptor (GR). After dimerization of GR, this complex acts as a transcription factor able to modify the expression of many genes [23, 24].

In the present study, we first show that hypoxia environment enhances the survival of a sub-group of leukemic cells specific to lymphoid B cells called B-ALL when treating with chemotherapies, such as MTX and PRD. The effect of hypoxia is not associated with an increase of total cell density nor cell proliferation. Then, using RPPA technology to screen death and survival signaling pathways we show a deregulation of pro- and anti-apoptotic pathways. Indeed, in the two B-ALL cell lines treated with MTX, hypoxia appears to inhibit the expression of pro-apoptotic proteins (Bax, Bim and Cleaved Caspase 3) and stimulate the expression of anti-apoptotic proteins (Bcl-2, Mcl-1). In the case of PRD, hypoxia seems to stimulate the expression of anti-apoptotic proteins (Bcl-2, Mcl-1 and XIAP). Results from western blotting experiments confirm that hypoxia is able to modulate the expression of anti-apoptotic proteins (Mcl-1 and Bcl-2). However, an up regulation of a pro-apoptotic protein (Bim) induced by a chemotherapy is not modulated by hypoxia.

Altogether, these results suggest that hypoxia can modulate the expression of pro and anti-apoptotic proteins allowing chemotherapy resistance in B-ALL cells and might play a role in patient relapse. Hypoxia, and to some extend HIF-1alpha, might represent a good therapeutic target for future drug development in addition to traditional chemotherapies. Indeed, a combined treatment of specific inhibitor of HIF-1alpha (P3155 or EZN-2968) with a classical chemotherapy could block the up-regulation of anti-apoptotic proteins pre-stimulated by the bone marrow environment and induce the over-expression of pro-apoptotic proteins.

## Methods

### Cell culture and treatments

The human leukemic cell lines Nalm-6 and Reh (DSMZ®, Braunschweig, Deutschland) were cultured in RPMI 1640 medium (Eurobio®, Courtaboeuf, France) containing 10 % fetal bovine serum (FBS, Eurobio®), 2 mM of L-glutamine (Eurobio®) with 5000 UI/L penicillin and 50 mg/L streptomycin (Eurobio®). Nalm-6 and Reh cell lines were maintained at 37 °C in a 5 % CO_2_ humidified atmosphere. Normoxic experiments at 37 °C were carried out under normal atmospheric conditions (21 % O_2_, 5 % CO_2_) while hypoxic experiments at 37 °C used a chamber (modified Anaerobic System Model 1029, Fisher Scientific®, Illkirch, France) giving 5 % O_2_, 5 % CO_2_, qs N_2_ (Air Products®, Paris, France). Methotrexate (MTX; (2S)-2-[[4-[(2,4-diaminopteridin-6-yl) methyl-methylamino] benzoyl]- amino] pentadioic acid) was obtained from a commercial source (Sigma-Aldrich®, Saint-Quentin Fallavier, France). Several concentrations were used 10 nM, 100 nM, 500 nM, 1 μM and 100 μM; two controls were added (culture media with or without solvent (NaCl 0.1 M). Prednisolone (PRD; Corticoïd) used for the study was from a commercial source (Sigma-Aldrich®). Several concentrations were used 10 nM, 100 nM, 1 μM, 10 μM and 100 μM; two controls were added: culture media with or without solvent (water).

### Validation of antibodies

Each candidate antibody was subjected to a stringent validation procedure before being certified for a use in RPPA. The antibodies had to have an analyte specific single band in Western blot against our cell line without non-specific binding. Antibodies selected: All antibodies mentioned here were validated by immunoblotting: Akt; P-Akt; Atg 3; Atg 5; Bak; Bax; Beclin-1; Bim; Total Caspase 3; Cleaved Caspase 3; Total Caspase 7; Cleaved Caspase 7; Total Caspase 8; Cleaved Caspase 8; Total Caspase 9; Cleaved Caspase 9; Cyclophilin A; Total Erk; P-Erk; P-FADD; Fas; HIF-1alpha; LC3A; LC3B; MCL-1; Total mTOR; P-mTOR; Puma; PTEN; p53; Total SAPK; P-SAPK; XIAP (Ozyme®, Saint-Quentin en Yvelines, France); Bcl-2 (Dako®, Les Ullis, France); β-Actine (Sigma-Aldrich®); Ki-67 (Santa Cruz, Heidelberg, Allemagne). The secondary antibodies used for our study were labeled with a marker emitting in the near infrared (680 nm) (Li-Cor Biosciences®, Nebraska, USA).

### Cell survival measurement

Nalm-6 and Reh cells were seeded onto a 48-well plate at 1×10^6^/mL cells per well (BD Falcon^TM^, BD Biosciences®, Le Pont de Claix, France), stored under normoxic or hypoxic conditions for 24 h, then treated with different concentrations of MTX or PRD under the same conditions and harvested at 24, 48 and 72 h. Cells were subsequently treated with one of 2 solutions: propidium iodide to determine the number of non-viable cells; detergent to determine the total number of cells. The viable cell density (total cell number multiplied by percentage of viable cells) and percentage of viable cells were read with an ADAM series automatic cell counter (Labtech®, Palaiseau, France). All data from cell survival measurements are presented as the mean ± the standard error of the mean (S.E.M.) Significant differences were determined by two-way ANOVA with a Bonferroni post-test using GraphPad Prism version 5.0 for Windows (GraphPad Software®, California, USA). Significant differences for cell viability measurements were determined by a Student’s paired *t*-test using GraphPad Prism version 5.0 for Windows (GraphPad Software®).

### Cell proliferation measurement

Nalm-6 and Reh cells were loaded with carboxy-fluorescein diacetate succinimidyl ester (CFDA SE, Fisher Scientific®) and seeded onto a 24-well plates at 1 × 10^6^/mL cells per well (BD Falcon^TM^, BD Biosciences®), then stored 24 h under normoxic or hypoxic conditions before incubation with MTX or PRD over 1, 2, 3 and 6 days in the same a normoxic or hypoxic environment. Cell division was determined by monitoring CFDA SE using a FACSCalibur (BD Biosciences®). Regarding the loading of the cell with CFDA SE, briefly, leukemic cells were centrifuged to obtain cell pellets. Pellets were resuspended with phosphate buffer saline (PBS 1X, Eurobio®) containing 0.1 % of bovine serum albumin (BSA, Eurobio®, Courtaboeuf, France). 10 mM of freshly prepared CFDA SE in dimethylsulfoxide (DMSO, Sigma-Aldrich®) was then added to Nalm-6 and Reh cell suspension to obtain a final working concentration of 10 μM before incubation at 37 °C for 15 min. To quench the staining reaction, 5 volumes of ice-cold culture media were added directly to the cell suspension and left for 5 min on ice. Cells were centrifuged at 1500 rpm during 5 min and the pellets resuspended with fresh culture media a total of three times. After diffusion into the cytoplasm of the leukemic cells CFDA SE was measured with a flow cytometer with 488 nm excitation and the data analysed using FlowJo software (TreeStar, Oregon, USA, Version 9.6). Acquisition based on 10,000 events (cells) was performed for each analysis.

### Reverse phase protein array

#### Spotting

The method used in this study has been described previously [25] (Additional file 1: Protocol S1). Briefly, RPPA assay was performed on 1 mg/mL of protein lysed with 20 mM of Hepes (pH7.9, Sigma-Aldrich®), 1 mM of MgCl_2_ (Sigma-Aldrich®), 1 % of NP-40 substitute (VWR®, Fontenay-Sous-Bois, France), 0.5 % of Sodium cholate (Sigma-Aldrich®), 0.25 % of n-dodecyl-β-D-maltoside (VWR®), 1 mM of Sodium orthovanadate (Sigma-Aldrich®) and 50 mM of Sodium fluoride (Sigma-Aldrich®) containing freshly added protease inhibitors and phosphatase inhibitors (Fisher Scientifics®, Illkirch, France). Lysed protein was mixed with 4X printing buffer [250 mM Tris (Sigma-Aldrich®), 50 % (v/v) Glycerol (Sigma-Aldrich®), 4 % (v/v) SDS (Sigma-Aldrich®), 10 % (v/v) 2-mercaptoethanol (Sigma-Aldrich®), 0.1 % (v/v) Tween 20 (Sigma-Aldrich®) in ddH_2_0]. Protein samples were printed onto nitrocellulose-coated glass slides (Sartorius®, Aubagne, Germany) with a SpotBot® 3 arrayer (Arrayit Corporation®, California, USA) and stocked overnight at 4 °C.

#### Hybridization

Slides were blocked with 50 % Odyssey blocking buffer (Li-Cor Biosciences ®) in PBS 1x for 1 h. Slides were incubated for 2 h with primary antibodies diluted at 1:100 and subsequently washed four times for 5 min in PBS 1x with 0.1 % Tween-20. Next, slides were incubated with infrared-labeled secondary antibody diluted at 1:2000 for 1 h in the dark. Washing steps were performed as described above. All washing and incubation steps were carried out at room temperature with gentle shaking. Finally, slides were rinsed in water and air-dried at room temperature. Slides were scanned with an Innoscan 710-IR infrared microarray scanner (Innopsys®, Carbonne, France) with 10 μm resolution and wavelength 670 nm.

#### Analysis

Analysis was performed on Mapix Software (Innopsys®). Background and non-specific binding was subtracted from total signal intensity for each spot. RPPA data were expressed as a Z score and values below or above two standard deviations away from the mean were analyzed. For Heatmap representations, a hierarchical clustering (Ward method) was performed on an open source software R. The hierarchical clustering calculation was based on an Euclidean distance method.

##### Western blot

Western immunoblotting analyses were done using material from 5 × 10^5^ to 10 × 10^6^ cells. Briefly, cells were washed in PBS, harvested, and solubilized in aforementioned lysis buffer and kept on ice for 20 min. Lysates were centrifuged at 10,000 g for 15 min at 4 °C and stocked at −80 °C. Protein lysates were dosed with BCA protein assay kit (Thermo Scientific®, Illkirch, France, [26]. 30 μg of proteins were diluted in loading buffer, heated for 5 min at 95 °C, size-separated in a 4–20 % pre-cast polyacrylamide gel (Fisher Scientifics®), and transferred to nitrocellulose membranes (GE Healthcare®, Aulnay sous Bois, France). Membranes were blocked with aforemention blocking buffer, washed, and incubated with the previously mentioned antibodies in blocking buffer + 0.1 % of Tween-20 (Sigma-Aldrich®). Primary antibodies were used at 1:250 to 1:1000 dilutions. Infrared-coupled secondary antibodies (IR-Dye680, Li-Cor Biosciences®) were used at 1:5000 dilutions. Membranes are visualized and analyzed on the Odyssey imaging system from Li-Cor (Li-Cor Biosciences®).

## Results

### MTX and PRD reduce leukemic cells viability in normoxic conditions

**Fig. 1:**
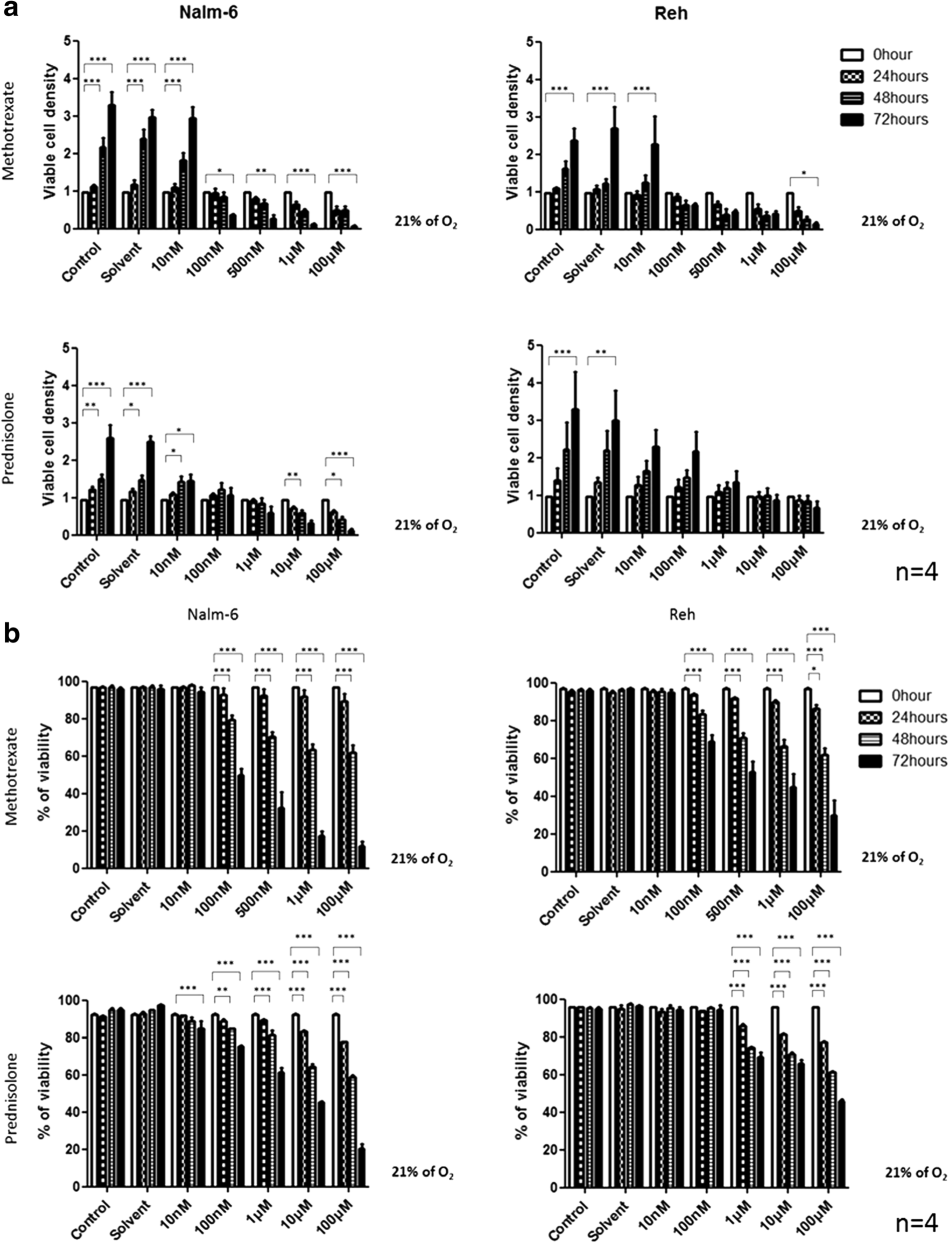
MTX and PRD inhibit leukemic cells viability in normoxia condition. **a** Effect of several concentrations of MTX or PRD over 72 h on viable cell density in normoxia (21 % O_2_) on leukemic cells. **b** Effect of several concentrations of MTX or PRD over 72 h on percentage of cell viability in normoxia (21 % O_2_) on leukemic cells. Viable cell density and percentage of cell viability measurement were performed by an automatic cell counter. All results are representatives with mean ± S.E.M. of 4 independent experiments. *indicates *p* < 0.05; **p < 0.01; ***p < 0.001 vs 0 h

In regard to the effect of MTX and PRD on leukemic B-ALL cells (Nalm-6 and Reh) cultured in normoxic conditions (21 % O_2_), viable cell density was significantly reduced in a time- and dose-dependent manner. Reh cells appear to be more resistant than Nalm-6 cells to both chemotherapies (Fig. 1a). A significant decrease in living cell density was observed for doses ≥ 100 nM MTX or 10 nM PRD in Nalm-6 cells and was mainly due to a decrease in cell viability compared to untreated cells (Fig. 1b). Reh viability was decreased by doses ≥ 100 nM MTX or 1 μM PRD (Fig. 1b) but living cell density was unaffected by PRD, suggesting that resistant cells maintain a proliferative activity even in the presence of high doses of corticoids (Fig. 1a).

### Hypoxia moderates the effect of MTX and PRD on leukemic cells without affecting viable cell density

**Fig. 2:**
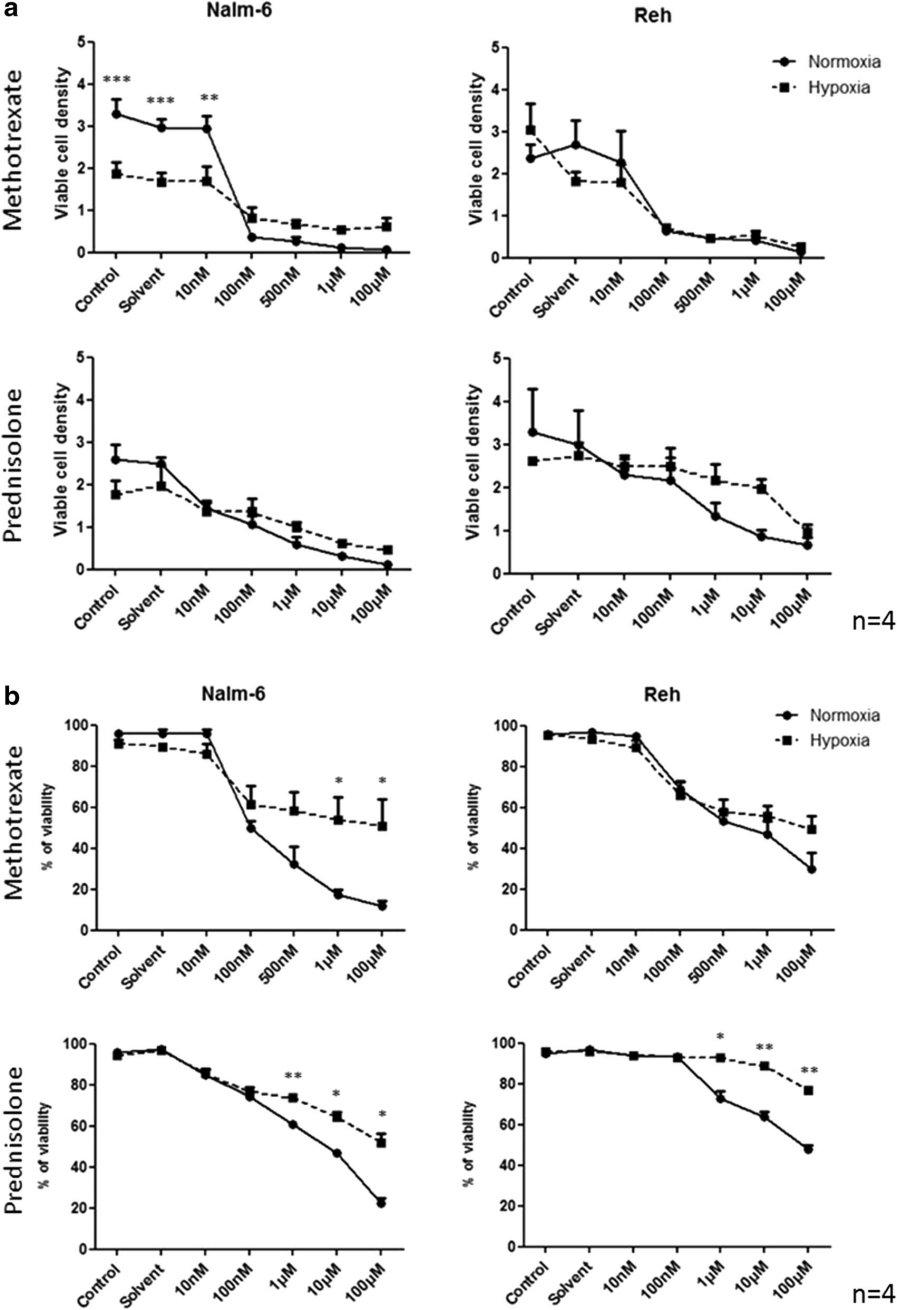
Hypoxia dampens MTX and PRD effect on leukemic cells viability without affecting viable cell density. **a** Effect of hypoxia (5 % O_2_) on viable cell density of leukemic cells treated with several concentrations of MTX or PRD at 72 h. **b** Effect of hypoxia (5 % O_2_) on viability of leukemic cells treated with several concentrations of MTX or PRD at 72 h. Viable cell density and percentage of cell viability measurement were performed by an automatic cell counter. All results are representatives with mean ± S.E.M. of 4 independent experiments. *indicates *p* < 0.05; **p < 0.01; ***p < 0.001 vs normoxia (21 % O_2_)

To investigate the effect of hypoxia on viable cell density and cell viability, Nalm-6 and Reh cells were cultured for 24 h under 5 % or 21 % O_2_ and then treated for 72 h with MTX or PRD at different concentrations. Nalm-6 and Reh cells exhibit the same sensitivity to MTX and PRD in both oxygenation conditions (Fig. 2a). However, in both cell lines, the decrease in cell viability was less pronounced under hypoxia than in normoxic conditions for PRD, and in the case of MTX for the Nalm-6 cell line (Fig. 2b). These observations suggest that hypoxia has a protective effect on leukemic cells that is not due to an increase in the number of viable cells. Furthermore, when checking leukemic cell proliferation, we observed no differences for different oxygen microenvironments in the absence of treatment (Additional file 2: Figure S1.a). After treatment with MTX, cell proliferation seems to be reduced in both cell lines while with PRD there is no effect (Additional file 2: Figure S1.b).

### Hypoxia inhibits chemotherapy-induced cell death pathways in leukemic cells

To understand how hypoxia can protect leukemic cells from cell death induced by chemotherapies, apoptotic pathways were screened by RPPA in both cell lines.

RPPA is an innovative technology allowing a screening of several molecular pathways on numerous samples. In this experiment, apoptosis pathways and proliferation / survival pathways were screened by RPPA. Nalm-6 and Reh cells were either untreated or treated with several concentrations of MTX (10nM – 100 μM) and PRD (10nM – 10 μM) over 24 h in both environments.

**Fig. 3:**
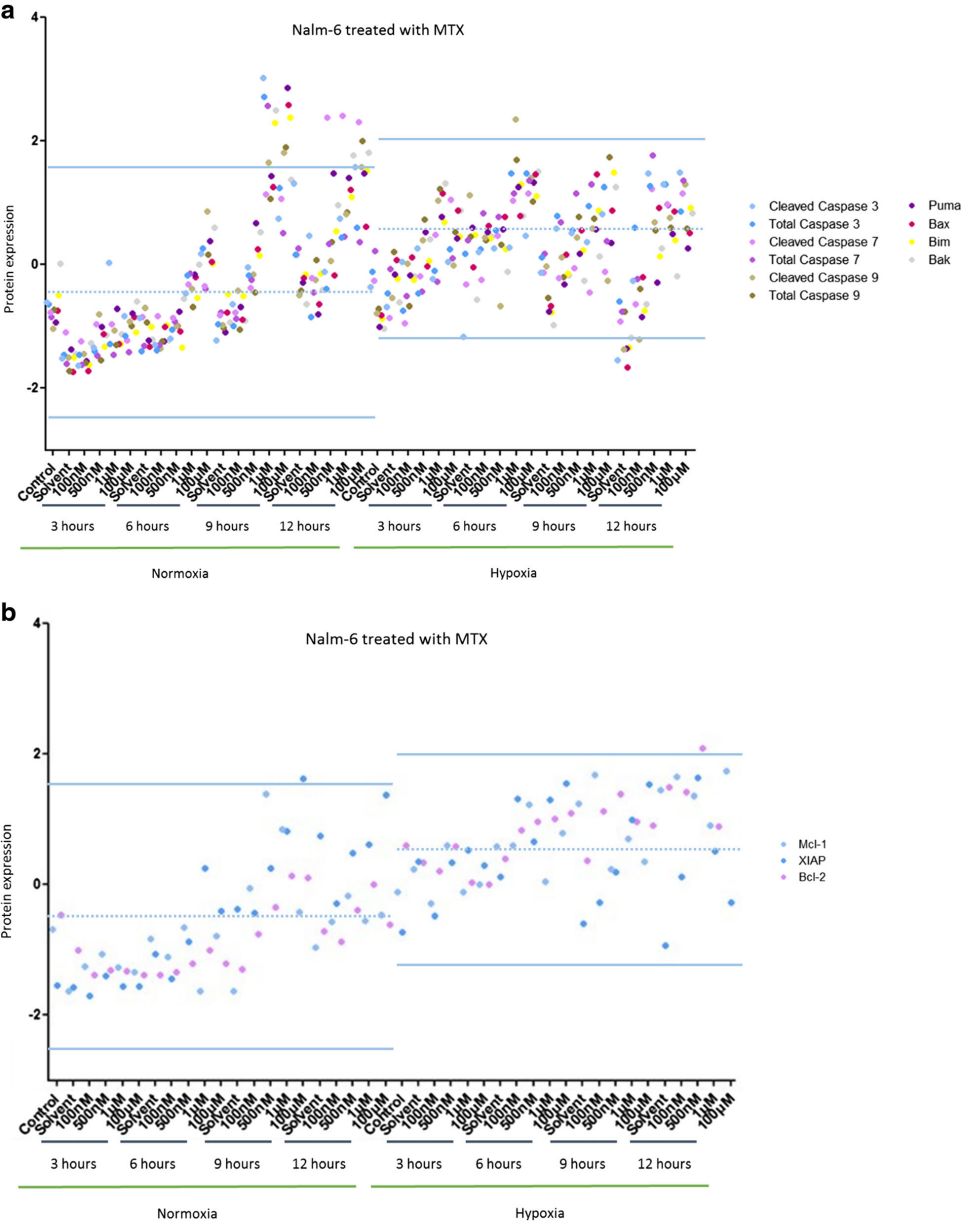
Hypoxia inhibits MTX-induced cell death pathways in Nalm-6 cells. **a** Effect of several concentrations of MTX over 24 h on Nalm-6 cells maintained in normoxia (21 % O_2_) or in hypoxia (5 % O_2_) on 10 pro-apoptotic proteins. **b** Effect of several concentrations of MTX over 24 h on Nalm-6 cells maintained in normoxia (21 % O_2_) or in hypoxia (5 % O_2_) on 3 anti-apoptotic proteins. Proteomic analysis was performed by RPPA. Data are represented by experimental conditions and by protein expressions after Z-score normalization. Protein expressions with two standard deviations away from the mean were analyzed

In Nalm-6 cells treated with MTX, a shift in protein expression profile was observed in hypoxia toward normoxia (Data not shown). In normoxia, the expression profile of pro-apoptotic proteins was increased in a dose- and time-dependent manner. Pro-apoptotic proteins (Cleaved Caspase 3, Bax and Bim) were over-expressed after MTX treatment in normoxic conditions (more than 2 standard deviations (SD) away from the mean) while in hypoxic conditions those three pro-apoptotic proteins were down-regulated (more than 2 SD away from the mean) (Fig. 3a). A shift in anti-apoptotic protein expression profile was observed in hypoxia toward normoxia (Fig. 3b).

**Fig. 4:**
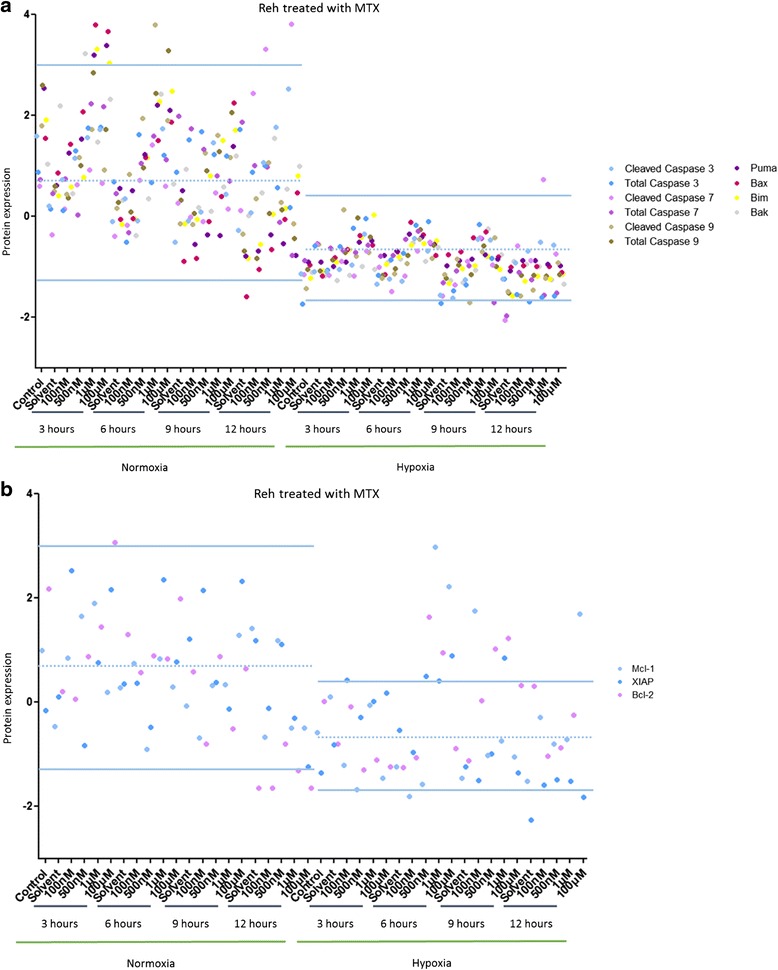
Hypoxia inhibits MTX-induced cell death pathways in Reh cells. **a** Effect of several concentrations of MTX over 24 h on Reh cells maintained in normoxia (21 % O_2_) or in hypoxia (5 % O_2_) on 10 pro-apoptotic proteins. **b** Effect of several concentrations of MTX over 24 h on Reh cells maintained in normoxia (21 % O_2_) or in hypoxia (5 % O_2_) on 3 anti-apoptotic proteins. Proteomic analysis was performed by RPPA. Data are represented by experimental conditions and by protein expressions after Z-score normalization. Protein expressions with two standard deviations away from the mean were analyzed

In Reh cells treated with MTX, a drop in protein expression was observed in hypoxia compared to normoxia (Data not shown). Pro-apoptotic proteins (Cleaved Caspase 7, Bax, Puma and Bim) were over-expressed after MTX treatment in normoxia (more than 2 SD away from the mean) (Fig. 4a). Anti-apoptotic proteins (XIAP, Mcl-1 and Bcl-2) were over-expressed after MTX treatment in hypoxia (more than 2 SD away from the mean) (Fig. 4b).

**Fig. 5:**
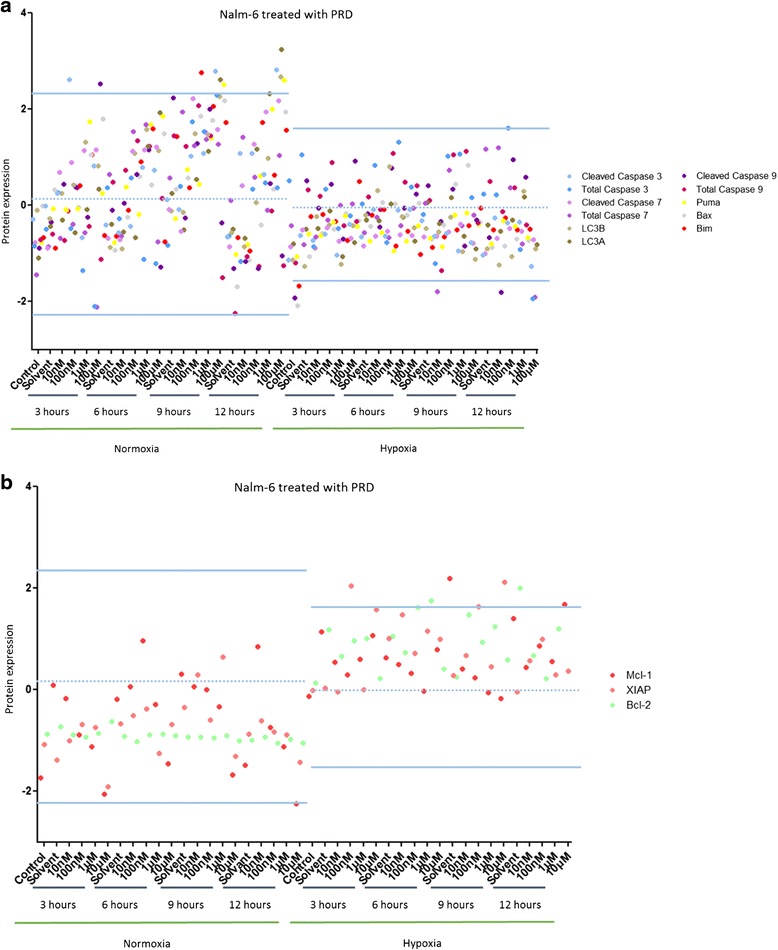
Hypoxia inhibits PRD-induced cell death pathways in Nalm-6 cells. **a** Effect of several concentrations of PRD over 24 h on Nalm-6 cells maintained in normoxia (21 % O_2_) or in hypoxia (5 % O_2_) on 11 pro-apoptotic and autophagy proteins. **b** Effect of several concentrations of PRD over 24 h on Nalm-6 cells maintained in normoxia (21 % O_2_) or in hypoxia (5 % O_2_) on 3 anti-apoptotic proteins. Proteomic analysis was performed by RPPA. Data are represented by experimental conditions and by protein expressions after Z-score normalization. Protein expressions with two standard deviations away from the mean were analyzed

In Nalm-6 cells treated with PRD, the protein expression profiles were unchanged by hypoxia and normoxia (Data not shown). Two pro-apoptotic proteins (cleaved Caspase 3 and Puma) and autophagy protein (LC3A) were over-expressed after PRD treatment in normoxia (more than 2 SD away from the mean) while in hypoxia three anti-apoptotic proteins (XIAP, Mcl-1 and Bcl-2) were up-regulated (more than 2 SD away from the mean) (Fig. 5a and b).

**Fig. 6:**
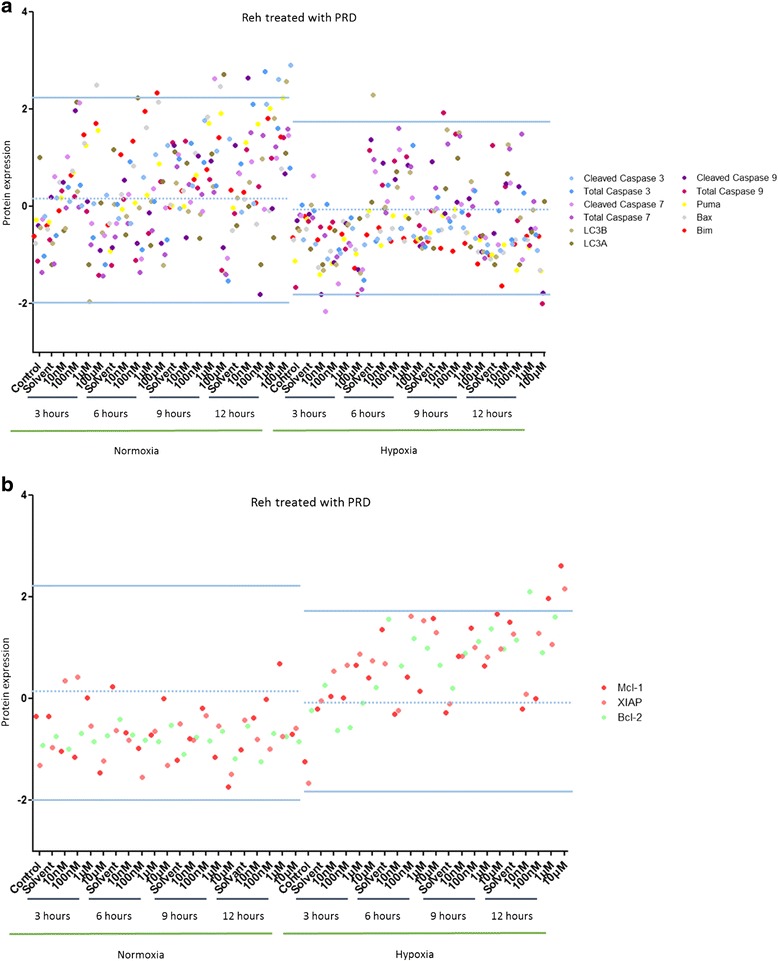
Hypoxia inhibits PRD-induced cell death pathways in Reh cells. **a** Effect of several concentrations of PRD over 24 h on Reh cells maintained in normoxia (21 % O_2_) or in hypoxia (5 % O_2_) on 11 pro-apoptotic and autophagy proteins. **b** Effect of several concentrations of PRD over 24 h on Reh cells maintained in normoxia (21 % O_2_) or in hypoxia (5 % O_2_) on 3 anti-apoptotic proteins. Proteomic analysis was performed by RPPA. Data are represented by experimental conditions and by protein expressions after Z-score normalization. Protein expressions with two standard deviations away from the mean were analyzed

In Reh cells treated with PRD, the protein expression profiles were unchanged by hypoxia and normoxia (Data not shown). In normoxia, at least three pro-apoptotic proteins (Bax, Bim and cleaved caspase 3) and two autophagy proteins (LC3A and LC3B) were up-regulated (more than 2 SD away from the mean) (Fig. 6a). The expression profile of anti-apoptotic proteins was markedly increased in hypoxia compared to normoxia (Fig. 6b).

To summarize, chemotherapies in normoxia induce an up-regulation of pro-apoptotic proteins while chemotherapies in hypoxia induce an up-regulation of anti-apoptotic proteins. All data point were also analyzed and represented in hierarchical clusters (Additional file 3: Figures S2, Additional file 4: Figures S3, Additional file 5: Figures S4, Additional file 6: Figures S5).

### Hypoxia promotes anti-apoptotic signals in leukemic cells independently of chemotherapies

**Fig. 7:**
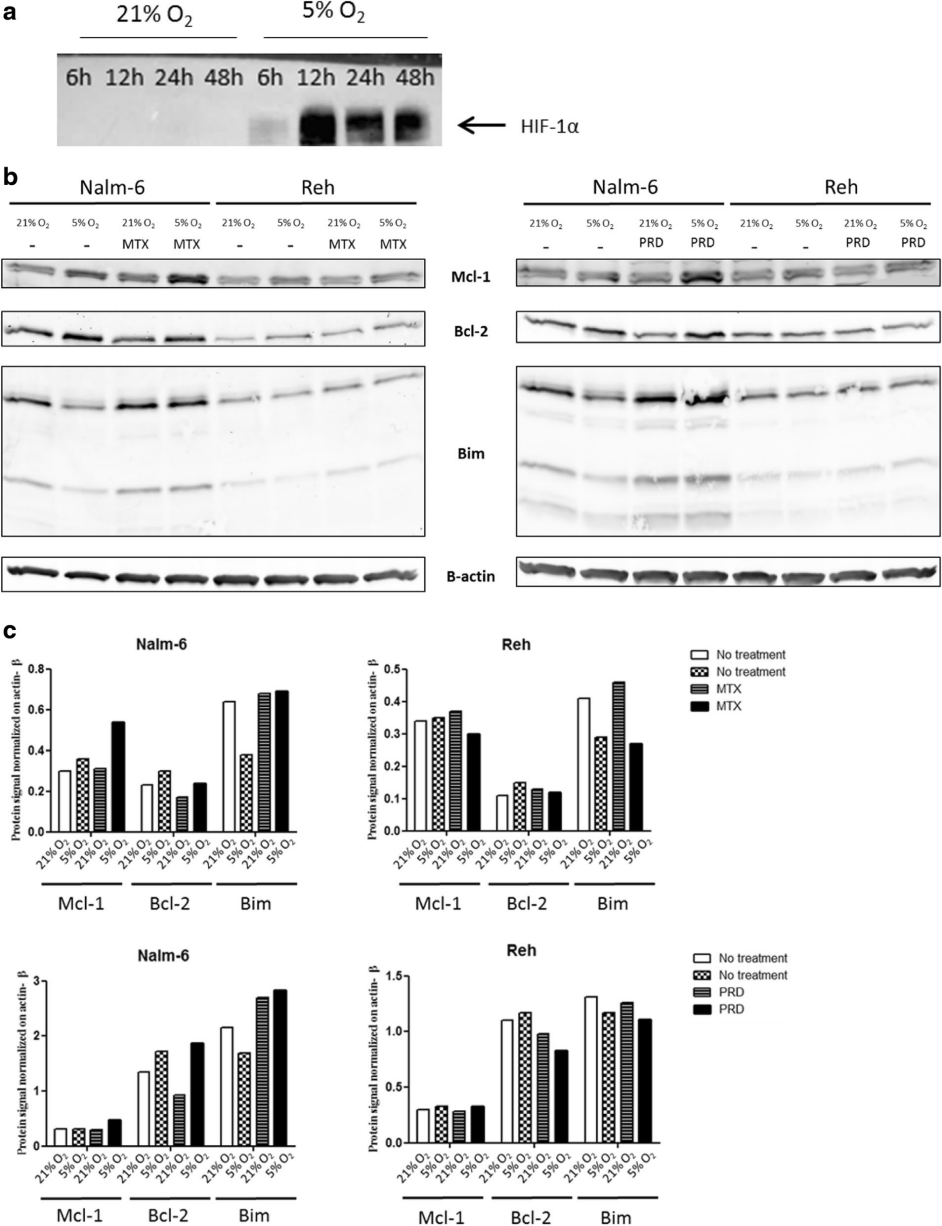
Hypoxia promotes anti-apoptotic signals in leukemic cells independently of chemotherapies. **a** Effect of hypoxia (5 % O_2_) over 48 h on nuclear expression of HIF-1α protein in Nalm-6 cells. **b** Nalm-6 and Reh cells were cultured either in normoxia or hypoxia and untreated or treated with MTX or PRD for 3 h and lysed. Equal amounts of proteins were fractionated by SDS-PAGE and Western blotted with anti-Mcl-1, anti-Bcl-2, anti-Bim and anti-β actin. **c** Western blotting quantification was performed on the image from Fig. 7B

To confirm the hypoxia condition in the experimental settings, HIF-1α expression (a classical marker of hypoxia) was analysed by western blotting on nuclear protein extract from Nalm-6 cells maintained either in normoxia or hypoxia environment over 48 h (Fig. 7a). An over-expression of HIF-1a was clearly observed after 6 h in hypoxia condition.

To confirm results obtained by RPPA, the expression of two anti-apoptotic proteins (Mcl-1 and Bcl-2) and one pro-apoptotic protein (Bim) were also studied in western blotting experiments. In untreated Nalm-6 cells, anti-apoptotic proteins (Mcl-1 and Bcl-2) were up-regulated in hypoxia. After MTX or PRD treatment, similar results were obtained in Nalm-6 cells in hypoxia (Fig. 7b). The pro-apoptotic protein Bim was decreased in untreated Nalm-6 cells in hypoxia compared to normoxia, while in MTX or PRD treated Nalm-6 cells no decrease in Bim expression was observed. Indeed, the intensity of signal was slightly increase compared to untreated Nalm-6 in normoxia (Fig. 7b). In untreated and MTX or PRD treated-Reh cells, the anti-apoptotic protein Mcl-1 was up-regulated in hypoxia while the other anti-apoptotic protein Bcl-2 has similar level of expression compared to normoxia (Fig. 7b). The expression of the pro-apoptotic protein Bim was not affected in untreated and MTX or PRD treated-Reh cells in hypoxia and normoxia (Fig. 7b).

Mcl-1 was up-regulated for both cell lines under hypoxia, without or with chemotherapies while Bcl-2 was up-regulated in hypoxia without or with chemotherapies in Nalm-6 cells only. Bim was down-regulated in hypoxia in the absence of chemotherapies in Nalm-6 cells only. In treated-Nalm-6 cells, Bim expression was increased compared to the untreated condition. In treated-Nalm-6 cells, Bim expression level was similar in both environments. These results were confirmed by western blotting quantification (Fig. 7c).

These results indicate that the increase in the expression of the anti-apoptotic proteins Mcl-1 and Bcl-2 is hypoxia dependent and chemotherapy independent. The data also show that the decrease in the expression of the pro-apoptotic protein Bim was hypoxia dependent in the absence of treatment while in the presence of treatment, the increase in the expression of Bim is not dampened by hypoxia.

## Discussion

Resistance to chemotherapy is associated with a bad prognosis in B-ALL and molecular mechanisms responsible for this resistance are poorly understood [27]. Indeed, drug resistance plays a crucial role in relapse of childhood ALL [28, 29]. MTX and PRD are two chemotherapies widely used in multi-drug treatment of leukemia [30]. Despite a clear and highly significant effect of these two molecules on childhood remission there is still relapse in 10 to 15 % of cases. Considering the importance of MTX and PRD in contemporary ALL-treatment protocols, elucidating the mechanisms involved in drug-resistance is of major clinical importance [31, 32]. MTX is a folic acid analog able to inhibit the *de novo* synthesis of purine and pyrimidine bases of DNA (DesoxyriboNucleic Acid) while PRD is a glucocorticoid able to regulate the transcription of numerous genes implicated in cell-cycle arrest and apoptosis of leukemic cells. Several studies have shown that a deregulation of protein expression could improve cancer cell survival after a chemical stress [33]. Protein expression modification can affect cell signaling pathways leading to alteration of the energy metabolism (glycolytic enzymes), ionic movement (calcium flux), cell motility (cytoskeletal proteins) and cell death mechanisms (apoptosis proteins) [34–36]. Others studies have shown that cancer cells could interact with the microenvironment [37, 38]. Nefedova et al. explains that microenvironment could alter the sensitivity of cancer cells to cytotoxic drugs or radiation [37]. This team shows that multiple interactions including cell-cell, cell-growth factor (soluble factors) and cell-extracellular matrix (molecular components and bone marrow environment) are able to influence cell survival. In leukemia, the interaction between cancer cells and microenvironment can lead to an improvement of cell survival and resistance to chemotherapies [39].

In hematological malignancies, leukemic cells have a strong interaction with BM microenvironment. Benito group has shown that the expansion of leukemic cells is increased in low O_2_ BM condition (hypoxia) [3]. Hypoxia plays a key role in BM microenvironment by modulating energy metabolism, angiogenesis and leukemic cell apoptosis. Only a few studies highlight the involvement of the microenvironment and low oxygen content in the deregulation of apoptotic process and resistance of leukemic blasts to chemotherapies. Within the BM, many hematopoietic niches provide a sanctuary for leukemic stem cells which evade chemotherapy-induced cell death and allow the acquisition of a drug-resistant phenotype [40]. Despite the well-established role of hypoxia in the acquisition of pro-survival properties and resistance to chemotherapies of ALL cells, the molecular mechanisms affected by hypoxia have not been completely elucidated [41]. It has been shown that the transcription factor hypoxia-inducible factor-1alpha (HIF-1alpha) is stabilized in hypoxic conditions and many participate in the inhibition of leukemic cell proliferation without promoting cell death. As shown in recent studies, hypoxia plays an important role in quiescence and the intrinsic properties of hematopoietic and leukemic stem cells [42, 43]. Frolova group also demonstrate that hypoxia can induce a resistance of ALL cell lines to several chemotherapies through a stabilization of HIF-1α. In our study, we have shown that a low level of O_2_ is able to induce leukemic cell resistance to chemotherapies (Fig. 2b).

Two hypothesis might explain this improvement of cell viability: an increase in cell proliferation or a better cell survival. We have found that leukemic cell proliferation measured by flow cytometry is not affected by hypoxia. To study cell survival, death signaling pathways were analyzed by RPPA. Cell death is part of the hematopoietic homeostasis. However, a deregulation of cell death mechanisms can disrupt the delicate equilibrium between cell proliferation, survival and death and can lead to the development of diseases (cancers, auto-immune diseases and neurodegenerative diseases). Several studies have shown that apoptotic pathway alterations could play a role in the induction of chemotherapy resistance in leukemia [44]. Testa group explain that in acute myeloid leukemia (AML) the alteration of apoptotic pathway with an induction of anti-apoptotic signals through p53 or Bcl-2 can promote survival of leukemic cells. Chetoui’s group demonstrated that Mcl-1, an anti-apoptotic protein from the Bcl-2 family that is regulated by extracellular signal-regulated kinases (ERK) signaling pathway, contributes significantly to the drug resistance of melanoma cells [45]. Furthermore, other studies show that overexpression of anti-apoptotic proteins such as inhibitor of apoptosis proteins (IAPs) may contribute to the development of cancer [46]. X-linked inhibitor of apoptosis protein (XIAP) is the best-defined of IAP family member able to neutralize directly the effector caspase 3. The XIAP protein level correlated with the sensitivity to multiple anti-cancer drugs. For example in AML, patients with lower levels of XIAP protein had a better survival rate and a tendency toward longer remission than those with higher levels of XIAP [47]. Our screening by RPPA has shown modification of apoptotic protein expression in hypoxia. Chemotherapies usually induce an increase of pro-apoptotic proteins (Bax, Bim and Caspase 3) and a decrease of anti-apoptotic proteins leading to cancer cell death. Surprisingly, in hypoxia, we observed an increase in anti-apoptotic signals (Bcl-2, Mcl-1 and XIAP) and a decrease in pro-apoptotic signals in drug-treated leukemic cell (Figs. 3 to 6). This observation was confirmed by RPPA and western blotting. This data shows that a low O_2_ content is able to directly modify cell death protein expression. Furthermore, an increase of anti-apoptotic proteins (Mcl-1 and Bcl-2) is already present in hypoxia in the absence of chemotherapies indicating a basal direct effect of hypoxia in favor of leukemic cell survival (Fig. 7). One hypothesis is that hypoxia is able to condition leukemic cells to therapeutic stress by tuning their intrinsic survival capacity. HIF-1alpha stabilized in hypoxia is a key factor in cancer cells and is associated with worse prognosis in many cancers. HIF-1alpha is a transcription factor able to induce the expression of death signaling pathway. Indeed, hypoxia has also been reported to inhibit apoptosis in several experimental conditions. The work of Dons et al. have shown that hypoxia could protect kidneys from staurosporine-induced apoptosis with an induction of IAP-2 protein [48]. Jin’s group have observed that hypoxia is able to prevent serum deprivation-induced apoptosis of tumor cells by decreasing the Bax/Bcl-2 ratio and inhibiting the caspase 3 activity [49]. As shown in our summary chart, we demonstrate that hypoxia potentiates leukemic cell resistance to chemotherapies (MTX and PRD) directly by induction of expression of multiple anti-apoptotic proteins such as Bcl-2 and Mcl-1 simultaneously (Table 1). This modification of protein expression is responsible for an increase in leukemic cells survival and could play a role in treatment failure.

**Table 1:** Summary chart of results obtained in this study

Nalm-6 no treatment						
	Cell density	Cell proliferation	Cell viability (%)	MCL-1 protein expression	Bcl-2 protein expression	Bim protein expression
Normoxa	More important	Equal	Equal	Weakly expressed	Weakly expressed	Overexpressed
Hypoxia	Less important	Equal	Equal	Overexpressed	Overexpressed	under expressed
Nalm-6 treated MTX						
	Cell density	Cell proliferation	Cell viability (%)	MCL-1 protein expression	Bcl-2 protein expression	Bim protein expression
Normoxa	inhibited by MTX	inhibited by MTX	inhibited by MTX	Overexpressed	Overexpressed	Equal
Hypoxia	inhibited by MTX	inhibited by MTX	Less inhibited	strongly expressed	strongly expressed	Equal
Nalm-6 treated PRD						
	Cell density	Cell proliferation	Cell viability (%)	MCL-1 protein expression	Bcl-2 protein expression	Bim protein expression
	inhibited by PRD	Equal	inhibited by PRD	Overexpressed	Overexpressed	Equal
	inhibited by PRD	Equal	Less inhibited	strongly expressed	strongly expressed	Equal
Reh no treatment						
	Cell density	Cell proliferation	Cell viability (%)	MCL-1 protein expression	Bcl-2 protein expression	Bim protein expression
Normoxia	More important	Equal	Equal	Overexpressed	Overexpressed	Equal
Hypoxia	Less important	Equal	Equal	strongly expressed	strongly expressed	Equal
Reh treated MTX						
	Cell density	Cell proliferation	Cell viability (%)	MCL-1 protein expression	Bcl-2 protein expression	Bim protein expression
Normoxia	inhibited by MTX	inhibited by MTX	inhibited by MTX	Overexpressed	Overexpressed	Equal
Hypoxia	inhibited by MTX	inhibited by MTX	inhibited by MTX	strongly expressed	strongly expressed	Equal
Reh treated PRD						
	Cell density	Cell proliferation	Cell viability (%)	MCL-1 protein expression	Bcl-2 protein expression	Bim protein expression
Normoxia	inhibited by PRD	Equal	inhibited by PRD	Overexpressed	Overexpressed	Equal
Hypoxia	inhibited by PRD	Equal	Less inhibited	strongly expressed	strongly expressed	Equal

## Conclusions

Our data clearly demonstrate an important role of low oxygen level in the response to treatment of leukemic cells. It appears that hypoxia is able to improve leukemic cell survival without modification of proliferation activities and that environmental conditions can modulate the protein expression of cancer cells. With our screening method of death signaling pathways, we demonstrate that hypoxia can increase the expression of anti-apoptotic proteins Mcl-1 and Bcl-2. This over-expression of anti-apoptotic proteins could contribute to the phenomenon of drug resistance of leukemic cells and treatment failure. Hypoxia and especially the increase in anti-apoptotic proteins Mcl-1 and Bcl-2 should be taken into account in the development of new chemotherapies and the design of new therapeutic strategies.

## Additional files

Additional file 1: Protocol S1. RPPA full protocol. (DOCX 15 kb) Additional file 2: Figure S1. Differential effect of MTX and PRD on leukemic cell proliferation independently of hypoxia. (A) Effect of hypoxia (5 % O2) on cell survival in leukemic cell (day 0, day 2 and day 6). (B) Effect of chemotherapies and hypoxia (5 % O2) on cell survival in leukemic cells (day 6). Nalm-6 and Reh cells were loaded with CFDA SE at day 0 and cultured either in normoxia or in hypoxia and in the presence of MTX or PRD. The relative decrease of CFDA SE staining was monitored by flow cytometry. (DOCX 111 kb) Additional file 3: Figure S2. RPPA analysis of Nalm-6 cell lines treated with MTX. Signal intensities were normalized by Z-score and signals were used for a hierarchical cluster analysis. A black color indicates that protein from Nalm-6 cells treated with several concentrations of MTX in normoxia versus hypoxia, matches the medium expression level calculated for a specific protein in a particular experimental condition. Higher level expression than mean is shown as a red color, and a green color refers to a lower level expression than mean. Protein names are listed below and experimental conditions are mentioned on the right-hand side. All antibodies used for RPPA were validated by Western blot. All antibodies are listed in Additional file 7: Table S1. (DOCX 145 kb) Additional file 4: Figure S3. RPPA analysis of Reh cell lines treated with MTX. Signal intensities were normalized by Z-score and signals were used for a hierarchical cluster analysis. A black color indicates that protein from Reh cells treated with several concentrations of MTX in normoxia versus hypoxia, matches the medium expression level calculated for a specific protein in a particular experimental condition. Higher level expression than mean is shown as a red color, and a green color refers to a lower level expression than mean. Protein names are listed below and experimental conditions are mentioned on the right-hand side. All antibodies used for RPPA were validated by Western blot. All antibodies are listed in Additional file 7: Table S1. (DOCX 144 kb) Additional file 5: Figure S4. RPPA analysis of Nalm-6 cell lines treated with PRD. Signal intensities were normalized by Z-score and signals were used for a hierarchical cluster analysis. A black color indicates that protein from Nalm-6 cells treated with several concentrations of PRD in normoxia versus hypoxia, matches the medium expression level calculated for a specific protein in a particular experimental condition. Higher level expression than mean is shown as a red color, and a green color refers to a lower level expression than mean. Protein names are listed below and experimental conditions are mentioned on the right-hand side. All antibodies used for RPPA were validated by Western blot. All antibodies are listed in Additional file 7: Table S1. (DOCX 150 kb) Additional file 6: Figure S5. RPPA analysis of Reh cell lines treated with PRD. Signal intensities were normalized by Z-score and signals were used for a hierarchical cluster analysis. A black color indicates that protein from Reh cells treated with several concentrations of PRD in normoxia versus hypoxia, matches the medium expression level calculated for a specific protein in a particular experimental condition. Higher level expression than mean is shown as a red color, and a green color refers to a lower level expression than mean. Protein names are listed below and experimental conditions are mentioned on the right-hand side. All antibodies used for RPPA were validated by Western blot. All antibodies are listed in Additional file 7: Table S1. (DOCX 148 kb) Additional file 7: Table S1. Antibody selected for RPPA-based targeted proteomics. (DOCX 204 kb)

## Abbreviations

ALL: Acute lymphoblastic leukemia
AML: Acute myeloid leukemia
BM: Bone marrow
CFDA SE: Carboxy-fluorescein diacetate succinimidyl ester
CO_2_: Carbon dioxide
DNA: Deoxyribonucleic acid
ERK: Extracellular signal-regulated kinases
FBS: Fetal bovine serum
GPR: Genepix results
HIF: Hypoxia inducible factor
HSC: Hematopoietic stem cell
IAPs: Inhibitor of apoptosis proteins
MTX: Methotrexate
O_2_: Oxygen
PBS: Phosphate buffered saline
PRD: Prednisolone
RPMI: Roswell Park Memorial Institute
RPPA: Reverse phase protein array
SD: Standard deviation
XIAP: X-linked inhibitor of apoptosis protein

## References

[1] Cui XY, Skretting G, Jing Y, Sun H, Sandset PM, Sun L (2013). Hypoxia influences stem cell-like properties in multidrug resistant K562 leukemic cells. Blood Cells Mol Dis.

[2] Liu X-W, Su Y, Zhu H, Cao J, Ding W-J, Zhao Y-C (2010). HIF-1α-dependent autophagy protects HeLa cells from fenretinide (4-HPR)-induced apoptosis in hypoxia. Pharmacol Res Off J Ital Pharmacol Soc.

[3] Benito J, Shi Y, Szymanska B, Carol H, Boehm I, Lu H (2011). Pronounced hypoxia in models of murine and human leukemia: high efficacy of hypoxia-activated prodrug PR-104. PLoS One.

[4] Rouault-Pierre K, Lopez-Onieva L, Foster K, Anjos-Afonso F, Lamrissi-Garcia I, Serrano-Sanchez M (2013). HIF-2α protects human hematopoietic stem/progenitors and acute myeloid leukemic cells from apoptosis induced by endoplasmic reticulum stress. Cell Stem Cell.

[5] Wilson WR, Hay MP (2011). Targeting hypoxia in cancer therapy. Nat Rev Cancer.

[6] Houghton PJ, Lock R, Carol H, Morton CL, Phelps D, Gorlick R (2011). Initial testing of the hypoxia-activated prodrug PR-104 by the pediatric preclinical testing program. Pediatr Blood Cancer.

[7] Frolova O, Samudio I, Benito JM, Jacamo R, Kornblau SM, Markovic A (2012). Regulation of HIF-1α signaling and chemoresistance in acute lymphocytic leukemia under hypoxic conditions of the bone marrow microenvironment. Cancer Biol Ther.

[8] Semenza GL (2011). Regulation of metabolism by hypoxia-inducible factor 1. Cold Spring Harb Symp Quant Biol.

[9] Keith B, Johnson RS, Simon MC (2012). HIF1α and HIF2α: sibling rivalry in hypoxic tumour growth and progression. Nat Rev Cancer.

[10] Ribera J-M, Oriol A (2009). Acute lymphoblastic leukemia in adolescents and young adults. Hematol Oncol Clin North Am.

[11] Kaspers GJL, Wijnands JJM, Hartmann R, Huismans L, Loonen AH, Stackelberg A (2005). Immunophenotypic cell lineage and in vitro cellular drug resistance in childhood relapsed acute lymphoblastic leukaemia. Eur J Cancer Oxf Engl.

[12] Szczepanek J, Styczyński J, Haus O, Tretyn A, Wysocki M (2011). Relapse of acute lymphoblastic leukemia in children in the context of microarray analyses. Arch Immunol Ther Exp.

[13] Stam RW, Den Boer ML, Schneider P, de Boer J, Hagelstein J, Valsecchi MG (2010). Association of high-level MCL-1 expression with in vitro and in vivo prednisone resistance in MLL-rearranged infant acute lymphoblastic leukemia. Blood.

[14] Wei G, Twomey D, Lamb J, Schlis K, Agarwal J, Stam RW (2006). Gene expression-based chemical genomics identifies rapamycin as a modulator of MCL1 and glucocorticoid resistance. Cancer Cell.

[15] Holleman A, Cheok MH, den Boer ML, Yang W, Veerman AJP, Kazemier KM (2004). Gene-expression patterns in drug-resistant acute lymphoblastic leukemia cells and response to treatment. N Engl J Med.

[16] Cheng X, Bennett RL, Liu X, Byrne M, Stratford MW (2013). PKR negatively regulates leukemia progression in association with PP2A activation, Bcl-2 inhibition and increased apoptosis. Blood Cancer J.

[17] Wang T-X, Shi X-Y, Cong Y, Wang S-G, Wang Y-Y, Zhang Z-Q (2013). Reversal of multidrug resistance by 5,5′-dimethoxylariciresinol-4-O-β-D-glucoside in doxorubicin-resistant human leukemia K562/DOX. Indian J Pharmacol.

[18] Huang Y-J, Frazier ML, Zhang N, Liu Q, Wei C (2014). Reverse-phase protein array analysis to identify biomarker proteins in human pancreatic cancer. Dig Dis Sci.

[19] Broutin S, Commo F, De Koning L, Marty-Prouvost B, Lacroix L, Talbot M (2014). Changes in signaling pathways induced by vandetanib in a human medullary thyroid carcinoma model, as analyzed by reverse phase protein array. Thyroid Off J Am Thyroid Assoc.

[20] Koufopantelis P, Georgakakou S, Kazanis M, Giaginis C, Margeli A, Papargiri S (2009). Direct injection liquid chromatography/positive ion electrospray ionization mass spectrometric quantification of methotrexate, folinic acid, folic acid and ondansetron in human serum. J Chromatogr B Analyt Technol Biomed Life Sci.

[21] De Beaumais TA, Jacqz-Aigrain E (2012). Intracellular disposition of methotrexate in acute lymphoblastic leukemia in children. Curr Drug Metab.

[22] Takimoto CH (1996). New antifolates: pharmacology and clinical applications. Oncologist.

[23] Kaspers GJ, Pieters R, Van Zantwijk CH, Van Wering ER, Van Der Does-Van Den Berg A, Veerman AJ (1998). Prednisolone resistance in childhood acute lymphoblastic leukemia: vitro-vivo correlations and cross-resistance to other drugs. Blood.

[24] Ariës IM, Jerchel IS, van den Dungen RESR, van den Berk LCJ, Boer JM, Horstmann MA (2014). EMP1, a novel poor prognostic factor in pediatric leukemia regulates prednisolone resistance, cell proliferation, migration and adhesion. Leukemia.

[25] Loebke C, Sueltmann H, Schmidt C, Henjes F, Wiemann S, Poustka A (2007). Infrared-based protein detection arrays for quantitative proteomics. Proteomics.

[26] Smith PK, Krohn RI, Hermanson GT, Mallia AK, Gartner FH, Provenzano MD (1985). Measurement of protein using bicinchoninic acid. Anal Biochem.

[27] Li Z, Koh GS, Lu Y, Kham SKY, Yeoh AEJ (2013). Vincristine and prednisolone combination reduces MDR1 and microenvironment-mediated treatment resistance in acute lymphoblastic leukemia. Blood.

[28] Bachmann PS, Gorman R, Papa RA, Bardell JE, Ford J, Kees UR (2007). Divergent mechanisms of glucocorticoid resistance in experimental models of pediatric acute lymphoblastic leukemia. Cancer Res.

[29] Klumper E, Pieters R, Veerman AJ, Huismans DR, Loonen AH, Hählen K (1995). In vitro cellular drug resistance in children with relapsed/refractory acute lymphoblastic leukemia. Blood.

[30] Ariës IM, Hansen BR, Koch T, van den Dungen R, Evans WE, Pieters R (2013). The synergism of MCL1 and glycolysis on pediatric acute lymphoblastic leukemia cell survival and prednisolone resistance. Haematologica.

[31] Silva KL, de Souza PS, Nestal de Moraes G, Moellmann-Coelho A, Vasconcelos F da C, Maia RC (2013). XIAP and P-glycoprotein co-expression is related to imatinib resistance in chronic myeloid leukemia cells. Leuk Res.

[32] Campana D, Coustan-Smith E, Manabe A, Buschle M, Raimondi SC, Behm FG (1993). Prolonged survival of B-lineage acute lymphoblastic leukemia cells is accompanied by overexpression of bcl-2 protein. Blood.

[33] Qinghong S, Shen G, Lina S, Yueming Z, Xiaoou L, Jianlin W (2015). Comparative proteomics analysis of differential proteins in respond to doxorubicin resistance in myelogenous leukemia cell lines. Proteome Sci.

[34] Yang H, Zhang Q, He J, Lu W (2010). Regulation of calcium signaling in lung cancer. J Thorac Dis.

[35] Belichenko I, Morishima N, Separovic D (2001). Caspase-resistant vimentin suppresses apoptosis after photodynamic treatment with a silicon phthalocyanine in jurkat cells. Arch Biochem Biophys.

[36] Zhai X, Lu J, Wang Y, Fang F, Li B, Gu W (2014). Reversal effect of bufalin on multidrug resistance in K562/VCR vincristine-resistant leukemia cell line. J Tradit Chin Med Chung Tsa Chih Ying Wen Pan Spons -China Assoc Tradit Chin Med Acad Tradit Chin Med.

[37] Nefedova Y, Landowski TH, Dalton WS (2003). Bone marrow stromal-derived soluble factors and direct cell contact contribute to de novo drug resistance of myeloma cells by distinct mechanisms. Leukemia.

[38] Iwamoto S, Mihara K, Downing JR, Pui C-H, Campana D (2007). Mesenchymal cells regulate the response of acute lymphoblastic leukemia cells to asparaginase. J Clin Invest.

[39] Kováč M, Vášková M, Petráčková D, Pelková V, Mejstříková E, Kalina T (2014). Cytokines, growth, and environment factors in bone marrow plasma of acute lymphoblastic leukemia pediatric patients. Eur Cytokine Netw.

[40] Matsunaga T, Takemoto N, Sato T, Takimoto R, Tanaka I, Fujimi A (2003). Interaction between leukemic-cell VLA-4 and stromal fibronectin is a decisive factor for minimal residual disease of acute myelogenous leukemia. Nat Med.

[41] Eliasson P, Rehn M, Hammar P, Larsson P, Sirenko O, Flippin LA (2010). Hypoxia mediates low cell-cycle activity and increases the proportion of long-term-reconstituting hematopoietic stem cells during in vitro culture. Exp Hematol.

[42] Takubo K, Goda N, Yamada W, Iriuchishima H, Ikeda E, Kubota Y (2010). Regulation of the HIF-1alpha level is essential for hematopoietic stem cells. Cell Stem Cell.

[43] Simon MC, Keith B (2008). The role of oxygen availability in embryonic development and stem cell function. Nat Rev Mol Cell Biol.

[44] Testa U, Riccioni R (2007). Deregulation of apoptosis in acute myeloid leukemia. Haematologica.

[45] Chetoui N, Sylla K, Gagnon-Houde J-V, Alcaide-Loridan C, Charron D, Al-Daccak R (2008). Down-regulation of mcl-1 by small interfering RNA sensitizes resistant melanoma cells to fas-mediated apoptosis. Mol Cancer Res MCR.

[46] Obexer P, Ausserlechner MJ (2014). X-linked inhibitor of apoptosis protein - a critical death resistance regulator and therapeutic target for personalized cancer therapy. Front Oncol.

[47] Tamm I, Kornblau SM, Segall H, Krajewski S, Welsh K, Kitada S (2000). Expression and prognostic significance of IAP-family genes in human cancers and myeloid leukemias. Clin Cancer Res Off J Am Assoc Cancer Res.

[48] Dong Z, Venkatachalam MA, Wang J, Patel Y, Saikumar P, Semenza GL (2001). Up-regulation of apoptosis inhibitory protein IAP-2 by hypoxia. Hif-1-independent mechanisms. J Biol Chem.

[49] Jin KL, Mao XO, Nagayama T, Goldsmith PC, Greenberg DA (2000). Induction of vascular endothelial growth factor and hypoxia-inducible factore-1alpha by global ischemia in rat brain. Neurosc.

