# Supplementary material for: Hypoxia promotes chemoresistance in acute lymphoblastic leukemia cell lines by modulating death signaling pathways
Source: BMC Cancer. 2016 Sep 22;16:746. doi: 10.1186/s12885-016-2776-1 (PMC5034444; doi:10.1186/s12885-016-2776-1)
Supplement: Additional file 1: Protocol S1. — RPPA full protocol. (DOCX 15 kb) [file 12885_2016_2776_MOESM1_ESM.docx]

**Reverse phase protein array (full protocol):**

**- Spotting:** The method used in this study has been described previously (25). Briefly, RPPA assay was done using material from 5 x 10^5^ to 2 x 10^6^ cells lysed with Biosims lysis buffer [20 mM of Hepes (pH7.9, Sigma-Aldrich®), 1 mM of MgCl_2_ (Sigma-Aldrich®), 1% of NP-40 substitute (VWR®, Fontenay-Sous-Bois, France), 0.5% of Sodium cholate (Sigma-Aldrich®), 0.25% of n-dodecyl-β-D-maltoside (VWR®), 1 mM of Sodium orthovanadate (Sigma-Aldrich®) and 50 mM of Sodium fluoride (Sigma-Aldrich®)] containing freshly added protease inhibitors and phosphatase inhibitors (Fisher Scientifics®, Illkirch, France). After protein quantification, protein samples were prepared in 384-well plate at 1.3 mg/mL in 7.5 µL of dosed-lysates. 2.5 µL of 4X printing buffer developed by Biosims [250 mM Tris (Sigma-Aldrich®), 50% (v/v) Glycerol (Sigma-Aldrich®), 4% (v/v) SDS (Sigma-Aldrich®), 10% (v/v) 2-mercaptoethanol (Sigma-Aldrich®), 0.1% (v/v) Tween 20 (Sigma-Aldrich®) in ddH_2_0] were added to obtain a final concentration of 1 mg/mL of total protein in each wells. Protein samples were printed onto nitrocellulose-coated glass slides (Sartorius®, Aubagne, Germany) with an automated robotic SpotBot® 3 arrayer (Arrayit Corporation®, California, USA) with a lateral distance = 1.8 mm, vertical distance = 1.6 mm, spot-to-spot distance = 350 µm, temperature = 25°C, and a relative humidity = 60 to 70%. This contact method allow ~0.5 nL of protein lysate to be transferred to the nitrocellulose glass slide per array pin touch. After the printing process, slides were stocked overnight at 4°C for a complete binding of protein to nitrocellulose glass slide.

**- Hybridization:** Slides were blocked with 50% of Odyssey blocking buffer (Li-Cor Biosciences ®) in PBS 1x for 1 hour at room temperature (RT). Primary antibodies were diluted at 1:100 dilutions in a solution of 50% Odyssey Blocking Buffer (LI-Cor Biosciences ®) in PBS 1x with 0.1 % of Tween-20. Slides were incubated for 2 hours at 4°C with primary antibodies and subsequently immersed in wash buffer (PBS 1x with 0.1% Tween-20) four times for 5 minutes. Next, slides were incubated with infrared-labeled secondary antibody at 1:2000 dilutions for 1 hour at RT in the dark. Washing steps were performed as described above. All washing and incubation steps were carried out at RT with gentle shaking. Finally, slides were rinsed in water and air-dried at room temperature. Slides were scanned with an Innoscan 710-IR infrared microarray scanner (Innopsys®, Carbonne, France) with 10 µm of resolution, wavelength at 670 nm. The images obtained are in TIFF format.

**- Analysis:** A plate file (indicating the type of samples in 384-well source plate) was created with Excel (Microsoft Corporation®, Washington, USA). Then, a Gal file was created with Arrayit Software (Arrayit Corporation®). This file lists all the coordinates of samples deposited on the coated-slide. Analysis of TIFF format image and the establishment of Gal file were performed on Mapix Software (Innopsys®). Mapix software generates a GPR (GenePix Results) file, background and non-specific binding from total signal intensity for each spots was subtracted. RPPA data were expressed as a Z score for normoxia and hypoxia respectively, and only values below or above two standard deviations away from the mean were analyzed. For Heatmap representations, a hierarchical clustering (Ward method) was performed on an open source software R. The hierarchical clustering calculation was based on an Euclidean distance method.
