# Supplementary figures and images for: Hypoxia promotes chemoresistance in acute lymphoblastic leukemia cell lines by modulating death signaling pathways
Source: BMC Cancer. 2016 Sep 22;16:746. doi: 10.1186/s12885-016-2776-1 (PMC5034444; doi:10.1186/s12885-016-2776-1)

Figure S1 :


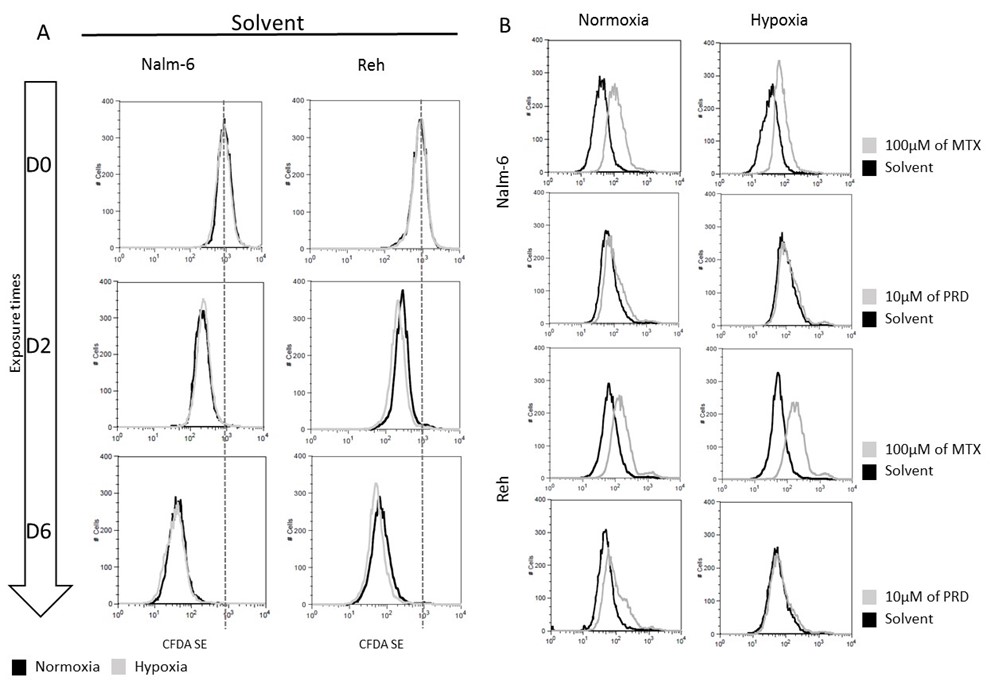

Supplement: Additional file 2: Figure S1. — Differential effect of MTX and PRD on leukemic cell proliferation independently of hypoxia. (A) Effect of hypoxia (5 % O2) on cell survival in leukemic cell (day 0, day 2 and day 6). (B) Effect of chemotherapies and hypoxia (5 % O2) on cell survival in leukemic cells (day 6). Nalm-6 and Reh cells were loaded with CFDA SE at day 0 and cultured either in normoxia or in hypoxia and in the presence of MTX or PRD. The relative decrease of CFDA SE staining was monitored by flow cytometry. (DOCX 111 kb) [file 12885_2016_2776_MOESM2_ESM.docx]

Figure S2 :


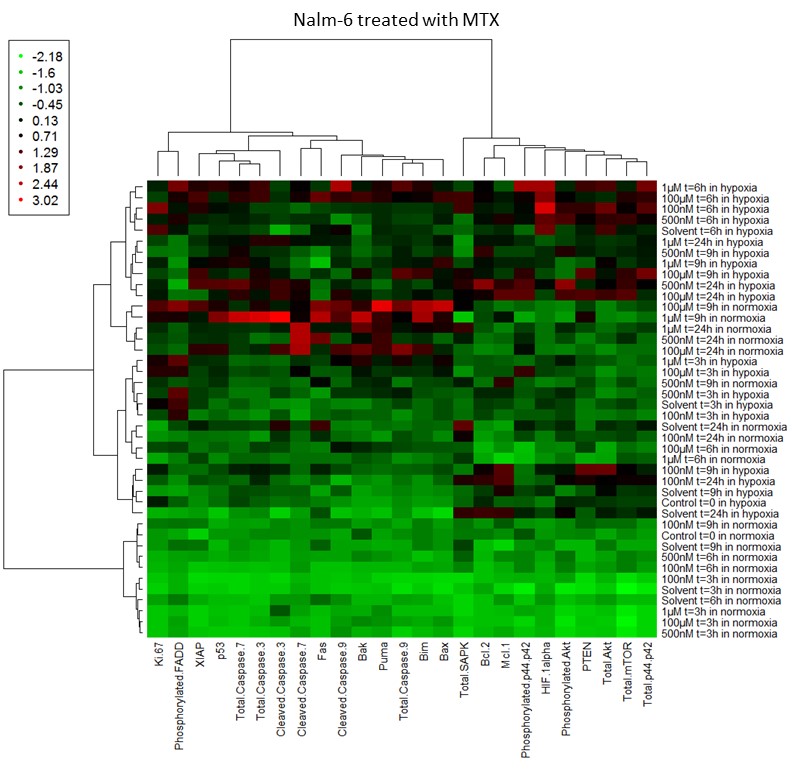

Supplement: Additional file 3: Figure S2. — RPPA analysis of Nalm-6 cell lines treated with MTX. Signal intensities were normalized by Z-score and signals were used for a hierarchical cluster analysis. A black color indicates that protein from Nalm-6 cells treated with several concentrations of MTX in normoxia versus hypoxia, matches the medium expression level calculated for a specific protein in a particular experimental condition. Higher level expression than mean is shown as a red color, and a green color refers to a lower level expression than mean. Protein names are listed below and experimental conditions are mentioned on the right-hand side. All antibodies used for RPPA were validated by Western blot. All antibodies are listed in Additional file 7: Table S1. (DOCX 145 kb) [file 12885_2016_2776_MOESM3_ESM.docx]

Figure S3 :


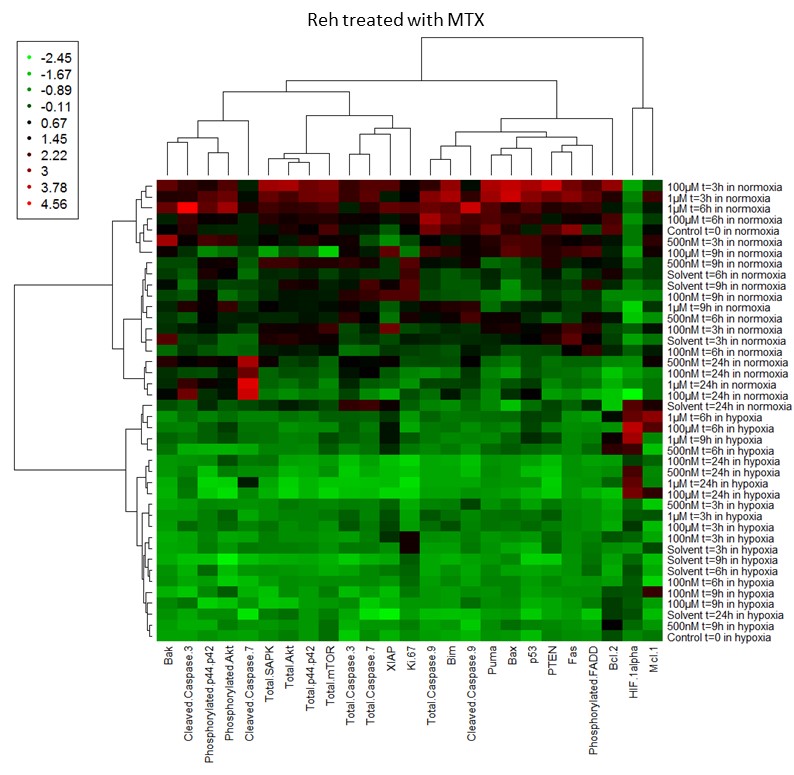

Supplement: Additional file 4: Figure S3. — RPPA analysis of Reh cell lines treated with MTX. Signal intensities were normalized by Z-score and signals were used for a hierarchical cluster analysis. A black color indicates that protein from Reh cells treated with several concentrations of MTX in normoxia versus hypoxia, matches the medium expression level calculated for a specific protein in a particular experimental condition. Higher level expression than mean is shown as a red color, and a green color refers to a lower level expression than mean. Protein names are listed below and experimental conditions are mentioned on the right-hand side. All antibodies used for RPPA were validated by Western blot. All antibodies are listed in Additional file 7: Table S1. (DOCX 144 kb) [file 12885_2016_2776_MOESM4_ESM.docx]

Figure S4 :


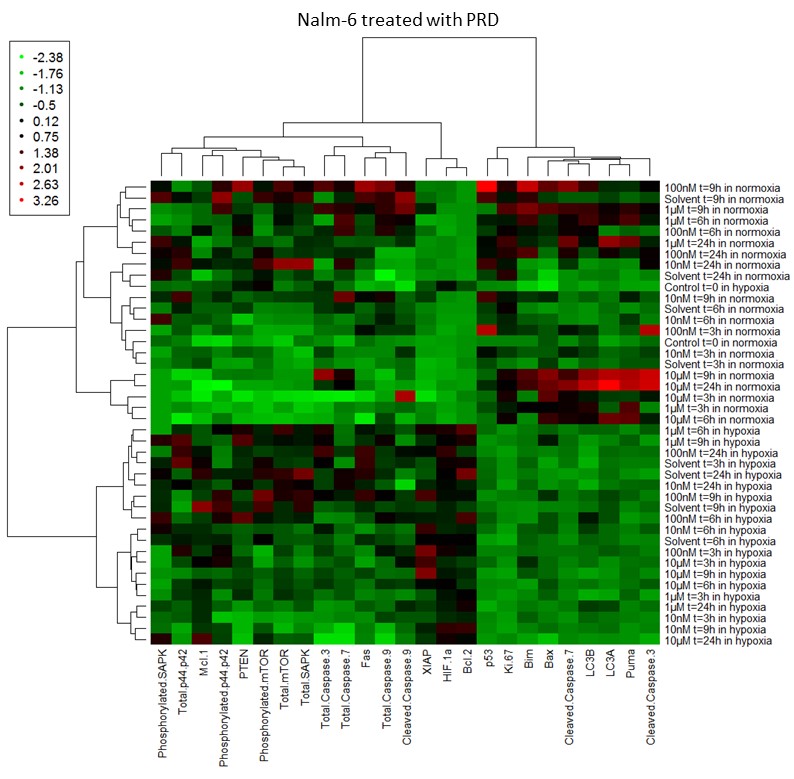

Supplement: Additional file 5: Figure S4. — RPPA analysis of Nalm-6 cell lines treated with PRD. Signal intensities were normalized by Z-score and signals were used for a hierarchical cluster analysis. A black color indicates that protein from Nalm-6 cells treated with several concentrations of PRD in normoxia versus hypoxia, matches the medium expression level calculated for a specific protein in a particular experimental condition. Higher level expression than mean is shown as a red color, and a green color refers to a lower level expression than mean. Protein names are listed below and experimental conditions are mentioned on the right-hand side. All antibodies used for RPPA were validated by Western blot. All antibodies are listed in Additional file 7: Table S1. (DOCX 150 kb) [file 12885_2016_2776_MOESM5_ESM.docx]

Figure S5 :


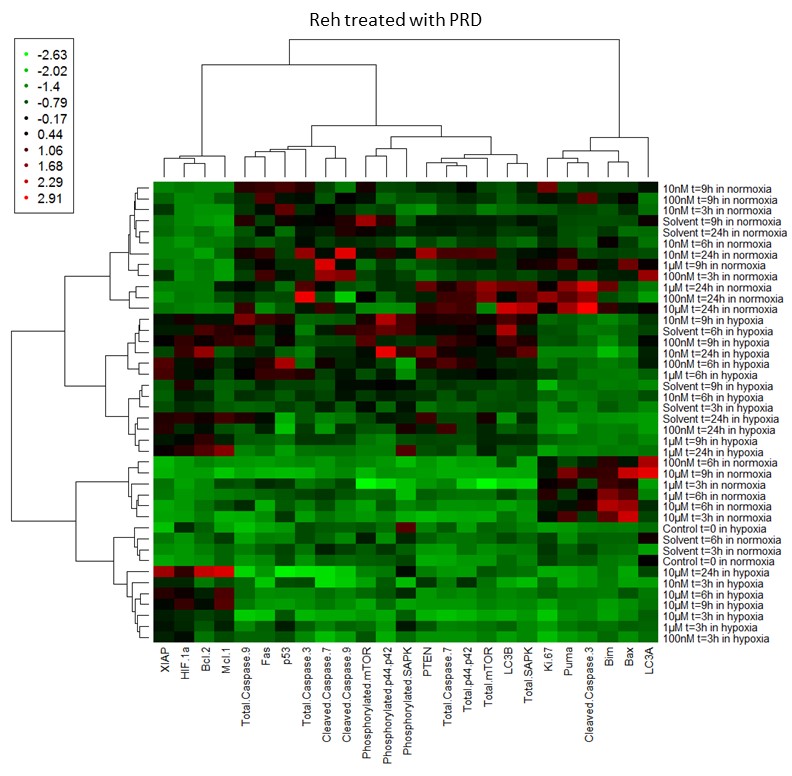

Supplement: Additional file 6: Figure S5. — RPPA analysis of Reh cell lines treated with PRD. Signal intensities were normalized by Z-score and signals were used for a hierarchical cluster analysis. A black color indicates that protein from Reh cells treated with several concentrations of PRD in normoxia versus hypoxia, matches the medium expression level calculated for a specific protein in a particular experimental condition. Higher level expression than mean is shown as a red color, and a green color refers to a lower level expression than mean. Protein names are listed below and experimental conditions are mentioned on the right-hand side. All antibodies used for RPPA were validated by Western blot. All antibodies are listed in Additional file 7: Table S1. (DOCX 148 kb) [file 12885_2016_2776_MOESM6_ESM.docx]

Table S1 :


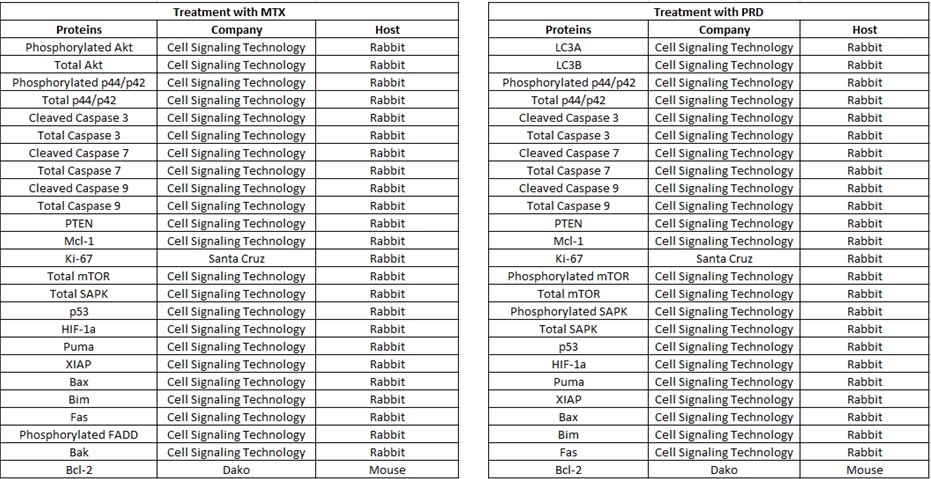

Supplement: Additional file 7: Table S1. — Antibody selected for RPPA-based targeted proteomics. (DOCX 204 kb) [file 12885_2016_2776_MOESM7_ESM.docx]
